# Association between changes in renal function and clinical outcomes in anticoagulated atrial fibrillation patients with marginal renal function. A nationwide observational cohort study

**DOI:** 10.3389/fcvm.2024.1423336

**Published:** 2024-06-06

**Authors:** Kyung-Yeon Lee, So-Ryoung Lee, Eue-Keun Choi, JungMin Choi, Hyo-Jeong Ahn, Soonil Kwon, Bongseong Kim, Kyung-Do Han, Seil Oh, Gregory Y. H. Lip

**Affiliations:** ^1^ Department of Internal Medicine, Seoul National University Hospital, Seoul, Republic of Korea; ^2^ Department of Internal Medicine, Seoul National University College of Medicine, Seoul, Republic of Korea; ^3^ Division of Cardiology, Department of Internal Medicine, Boramae Medical Center, Seoul National University College of Medicine, Seoul, Republic of Korea; ^4^ Statistics and Actuarial Science, Soongsil University, Seoul, Republic of Korea; ^5^ Liverpool Centre for Cardiovascular Science at University of Liverpool, Liverpool John Moores University and Liverpool Chest & Heart Hospital, Liverpool, United Kingdom; ^6^ Danish Center for Health Services Research, Department of Clinical Medicine, Aalborg University, Aalborg, Denmark

**Keywords:** atrial fibrillation, marginal renal function, renal function change, anticoagulation, renal dysfunction

## Abstract

**Background:**

Renal function is one of the crucial components for determining the dose and type of oral anticoagulants in atrial fibrillation (AF) patients, and is also closely associated with the risks of stroke and bleeding. This study aimed to assess renal function changes and their impact on clinical outcomes in anticoagulated AF patients with marginal renal function.

**Methods:**

From a Korean claims database, patients with AF on anticoagulants and a baseline eGFR of 45 to <60 ml/min/1.73 m^2^ were studied. Patients were grouped by changes in renal function over two years—maintained, improved (eGFR >60 ml/min/1.73 m^2^), or worsened (eGFR <45 ml/min/1.73 m^2^)—the study analyzed outcomes including ischemic stroke, major bleeding, end-stage renal disease (ESRD), all-cause death, and a composite of clinical outcomes.

**Results:**

A total of 5,126 patients were included in the study: 2,170 (42.3%) in the maintained group, 2,276 (44.4%) in the improved group, and 680 (13.1%) in the group with worsened renal function. The worsened group was older and had more prevalent comorbidities than other groups. After multivariable adjustment, the worsened group was associated with significantly higher risks of major bleeding (adjusted hazard ratio, 95% confidence interval; 1.46, 1.03–2.07, *p* = 0.035), ESRD (1.49, 1.24–1.80, *p* < 0.001), all-cause death (9.29, 4.92–17.6, *p* < 0.001), and the composite outcome (1.57, 1.36–1.83, *p* < 0.001).

**Conclusions:**

In anticoagulated AF patients with marginal renal function, a substantial proportion of patients experienced renal function decline below eGFR 45 ml/min/1.73 m^2^ within 2 years. Renal function decline was associated with higher risks of major bleeding, ESRD, all-cause death, and the composite outcome compared to those who maintained their baseline renal function.

## Introduction

Patients with atrial fibrillation (AF) commonly have associated renal dysfunction, with approximately 30%–60% of AF patients having mild to moderate renal dysfunction, and 3% of AF patients having severe renal dysfunction (1, 2). As stroke prevention is one of the pillars of AF management, oral anticoagulants (OACs) are recommended in guidelines (3), but those with impaired renal function require special attention due to their higher risk of stroke as well as bleeding (4–6).

In previous studies, AF patients with renal dysfunction are associated with a higher risk of stroke, bleeding, and death (2, 6). Furthermore, direct oral anticoagulants (DOACs) are the preferred OAC option but DOACs are excreted through the kidneys and the extent of excretion varies based on kidney function, with implications for DOAC dose adjustments (1).

In clinical practice, an eGFR of 45 to <60 ml/min/1.73 m^2^ (CKD stage 3a) is regarded as marginal renal function due to the clinical significance associated with stage 3a; indeed, CKD stage 3b or higher is recognized as a risk factor for the progression to end-stage renal disease (ESRD) (7, 8). Therefore, CKD stage 3a has clinical implications for implementing appropriate monitoring and preserving renal function to achieve better outcomes, especially since renal function dynamically changes with advancing age and clinical conditions such as dehydration, acute kidney injury, and cardiorenal syndrome. Hence, it is essential to actively monitor renal function (9) in order to prevent DOAC overdosing or underdosing, which could lead to an increased risk of clinical outcomes (10–12). However, the occurrence of renal function changes (whether improvement or worsened) in large population cohorts and its impact on clinical outcomes are not well-established in anticoagulated AF patients with marginal renal function.

This study aimed to evaluate renal function changes during follow-up in anticoagulated AF patients with marginal renal function, defined as an estimated glomerular filtration rate (eGFR) of 45 to <60 ml/min/1.73 m^2^. Second, we analyzed the associations between renal function changes and clinical outcomes.

## Methods

### Study participants

This study was a nationwide observational cohort study that utilized data sourced from a Korean nationwide claims database. The database acquired clinical details of Korean residents through routine national health examinations administered by the Korean National Health Insurance Service (NHIS). Participation in the NHIS is obligatory for all Korean citizens, with regular health assessments recommended annually or biennially (13, 14). AF patients underwent national health check-ups from January 2014 to December 2018 (first health examination) and had follow-up examination within 2-year (second health examination) were initially selected. We then included patients who were taking OAC therapy including warfarin, apixaban, dabigatran, edoxaban, and rivaroxaban and whose eGFR level was 45 to <60 ml/min/1.73 m^2^ (stage 3a) at baseline examination (first health examination). We excluded participants with the following: (1) aged under 20 years, (2) valvular AF, (3) alternative indications for OAC such as those receiving joint replacement surgery, with a history of pulmonary embolism, or a history of deep vein thrombosis, (4) without laboratory test results within the four years preceding the initiation of anticoagulation, (5) patients who were diagnosed with ESRD, were on dialysis, and underwent kidney transplantation at baseline, and (6) missing values in the baseline variables.

**Figure 1:**
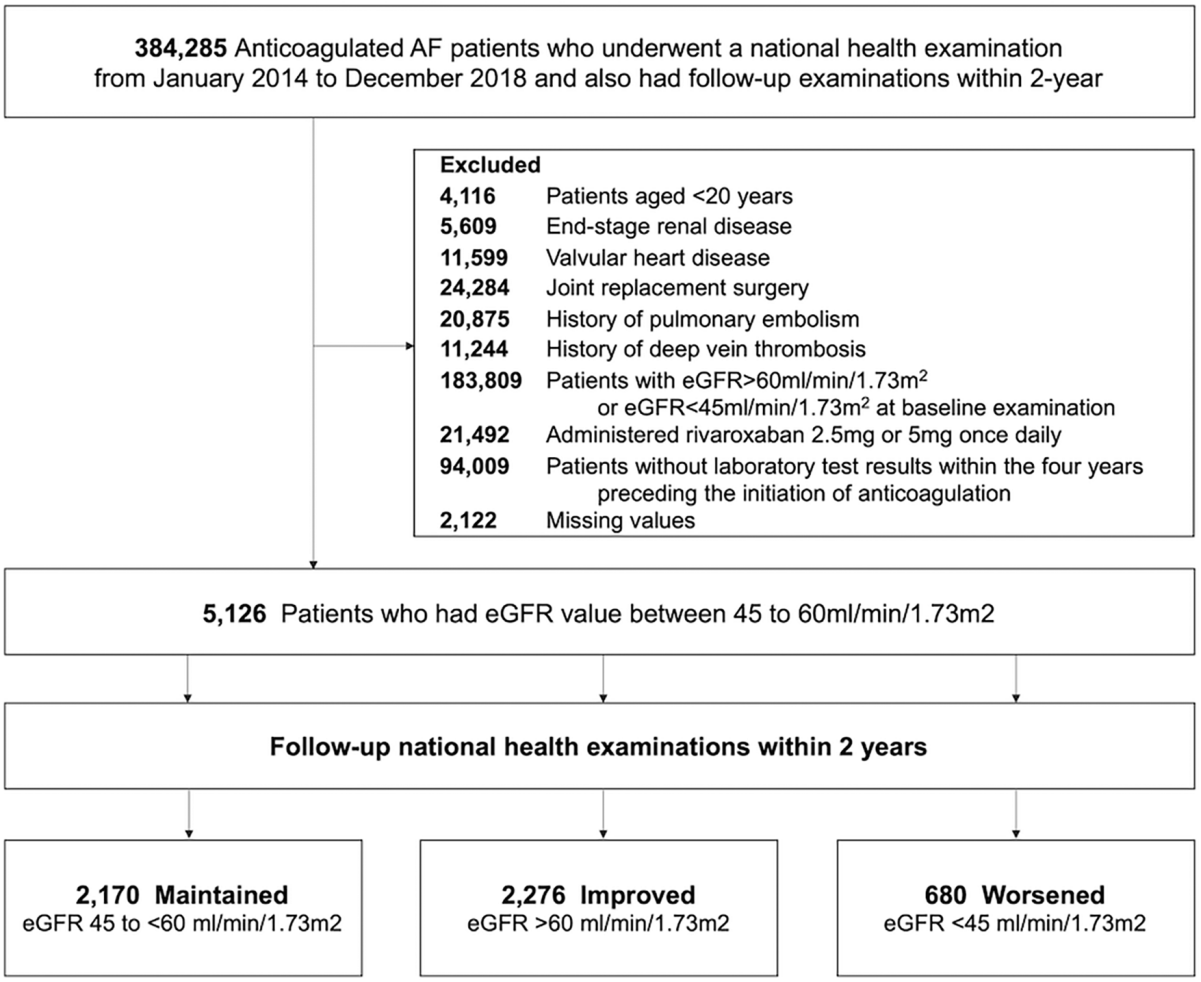
Study enrollment flow. AF, atrial fibrillation; eGFR, estimated glomerular filtration rate.

Based on the follow-up eGFR values at the second health examination, patients were categorized into three groups according to their change in renal function: maintained group (maintaining follow-up eGFR ranges from 45 to <60 ml/min/1.73 m^2^), improved group (eGFR >60 ml/min/1.73 m^2^), and worsened group (eGFR <45 ml/min/1.73 m^2^). The flow diagram of the present study is shown in Figure 1. The study was approved by the Institutional Review Board of Seoul National University Hospital (E-2309-095-1467); the necessity for obtaining informed consent was exempted since personal identifying details were removed while forming a cohort in adherence to rigorous confidentiality protocols.

### Covariates

The detailed definitions of covariates and outcomes used in the present study are shown in Supplementary Table S1. The patient's clinical data, including age, sex, physical measurements such as body weight and body mass index (BMI), as well as levels of serum creatinine (SCr) and estimated glomerular filtration rate (eGFR), were acquired from the baseline health examination results. The covariates of comorbidities include hypertension, diabetes mellitus, dyslipidemia, heart failure, prior ischemic stroke, prior MI, prior stroke, prior intracranial hemorrhage, prior gastrointestinal bleeding, peripheral artery disease, liver disease, chronic obstructive pulmonary disease (COPD), and cancer. The CHA_2_DS_2_-VASc score, HAS-BLED score, and Charlson comorbidity index (CCI) were calculated based on the baseline covariates, comorbidities, and medical history of the patients (Supplementary Tables S1 and S2). The information about antiplatelets (aspirin, P2Y12 inhibitor, or both) and OACs (warfarin, apixaban, dabigatran, edoxaban, or rivaroxaban) was acquired from the prescription records. The standard doses for DOACs were specified as follows: apixaban 5 mg twice daily, dabigatran 150 mg twice daily, edoxaban 60 mg once daily, and rivaroxaban 20 mg once daily. Low doses of DOACs were defined as apixaban 2.5 mg twice daily, dabigatran 110 mg twice daily, edoxaban 30 mg once daily, and rivaroxaban 15 mg once daily. Furthermore, very low doses of DOACs were defined as edoxaban 15 mg once daily and rivaroxaban 10 mg once daily. Dose labeling followed the criteria for dose reduction defined in four randomized controlled trials (9).

### Study outcomes and follow-up

The primary outcome was the *composite clinical outcome*, defined as the combination of ischemic stroke, major bleeding, incident kidney failure, and all-cause death. Kidney failure was defined as the need for maintenance dialysis or having kidney transplantation (Supplementary Table S1). The secondary outcomes were ischemic stroke, major bleeding, kidney failure, all-cause death, the composite of stroke and major bleeding, and the composite of stroke, major bleeding, and all-cause death. Detailed definitions of clinical outcomes are shown in Supplementary Table S1. The study evaluated clinical outcomes from the second health examination until the first occurrence of the index outcome, death, or until the end of the study (December 31, 2018), whichever occurred first.

### Statistical analysis

Continuous variables were expressed as means ± standard deviations, while categorical variables were presented as absolute values (percentages). To assess the significance of differences among groups, a one-way analysis of variance was used for continuous variables, and the chi-square test was employed for categorical variables. The incidence rates (IR) of outcomes were calculated using the Kaplan–Meier method and reported as the number of events per 100 person-years. Hazard ratios (HRs) and their corresponding 95% confidence intervals (CIs) were determined using Cox proportional hazard regression models. The multivariable model was utilized to calculate adjusted HRs and CIs for the covariates. Unadjusted HRs (Model 1); and HRs adjusted for age, sex, CHA_2_DS_2_-VASc score, HAS-BLED score, CCI, hypertension, diabetes mellitus, dyslipidemia, heart failure, prior myocardial infarction (MI), prior ischemic stroke, prior intracranial hemorrhage (ICH), prior gastrointestinal bleeding (GIB), peripheral artery disease, Liver disease, COPD, cancer, body weight, eGFR, antiplatelet (Model 2) were assessed serially. Statistical significance was defined at a two-tailed *p*-value <0.05. SAS version 9.4 was used to conduct the statistical analyses (SAS Institute, Cary, NC).

## Results

A total of 5,126 patients were finally included in this analysis. Among these, 42.3% of patients (*n* = 2,170) maintained their renal function, 44.4% (*n* = 2,276) showed improved renal function, while 13.3% (*n* = 680) showed worsened renal function at follow-up health examination. The mean follow-up durations for the primary outcome in each group were 3.7 ± 1.3, 3.8 ± 1.3, and 3.3 ± 1.5 years, respectively.

### Baseline characteristics according to the renal function changes

Baseline characteristics according to the renal function changes were presented in Table 1. The worsened group had a higher proportion of individuals over 75 years old, while the improved group had a higher proportion of people under 65. The worsened group had a significantly higher proportion of low body weight subjects, as well as higher mean CHA_2_DS_2_-VASc scores and more prevalent comorbidities including hypertension, heart failure, prior MI, peripheral artery disease, and COPD. The worsened group had significantly higher baseline serum creatinine levels and lower eGFR than the maintained group and improved group. At the second health examination, the mean eGFR values of maintained, improved, and worsened groups were 53.2 ± 4.3, 76.2 ± 46.4, and 37.6 ± 6.4 ml/min/1.73 m^2^, respectively (*p* < 0.001).

**Table 1:** Baseline characteristics according to renal function changes.

	Maintained (*n* = 2,170)	Improved (*n* = 2,276)	Worsened (*n* = 680)	*p*-value
Interval of medical check-ups, years, mean SD	2.23 ± 0.84	2.28 ± 0.88	2.39 ± 0.88	<0.001
Age, years	71.87 ± 7.16	70.08 ± 8.00	74.59 ± 7.03	<0.001
<65	305 (14.06)	492 (21.62)	65 (9.56)	<0.001
65 to <75	1,040 (47.93)	1,091 (47.93)	237 (34.85)	
≥75	825 (38.02)	693 (30.45)	378 (55.59)	
Men	1,190 (54.84)	1,291 (56.72)	373 (54.85)	0.404
CHA_2_DS_2_-VASc, mean SD	4.55 ± 1.62	4.27 ± 1.69	5.03 ± 1.62	<0.001
≥3	1,955 (90.09)	1,918 (84.27)	638 (93.82)	<0.001
CCI, mean SD	5.29 ± 2.67	497 ± 2.61	6.27 ± 3.00	<0.001
≥3	1,837 (84.65)	1,867 (82.03)	616 (90.59)	<0.001
HAS-BLED score^a^	2.64 ± 0.97	2.44 ± 0.96	3.07 ± 1.02	<0.001
Comorbidities				
Hypertension	2,038 (93.92)	2,092 (91.92)	660 (97.06)	<0.001
Diabetes mellitus	1,203 (55.44)	1,135 (49.87)	430 (63.24)	<0.001
Dyslipidemia	1,810 (83.41)	1,804 (79.26)	573 (84.26)	<0.001
Heart failure	1,169 (53.87)	1,189 (52.24)	421 (61.91)	<0.001
Prior ischemic stroke	507 (23.36)	529 (23.24)	167 (24.56)	0.768
Prior myocardial infarction	106 (4.88)	115 (5.05)	55 (8.09)	0.004
Prior intracranial hemorrhage	19 (0.88)	34 (1.49)	6 (0.88)	0.121
Prior gastrointestinal bleeding	148 (6.82)	147 (6.46)	65 (9.56)	0.019
Liver disease	1,077 (49.63)	1,148 (50.44)	374 (55)	0.047
Peripheral artery disease	709 (32.67)	695 (30.54)	249 (36.62)	0.010
COPD	170 (7.83)	181 (7.95)	81 (11.91)	0.002
Cancer	724 (33.36)	773 (33.96)	225 (33.09)	0.874
Antiplatelet Use				0.004
None	1,397 (64.38)	1,452 (63.8)	393 (57.79)	
Aspirin only	352 (16.22)	371 (16.3)	111 (16.32)	
P2Y12 only	181 (8.34)	203 (8.92)	65 (9.56)	
Both	240 (11.06)	250 (10.98)	111 (16.32)	
Baseline Body weight, mean SD	65.28 ± 11.23	65.43 ± 11.51	63.51 ± 11.3	<0.001
<50 kg	162 (7.47)	176 (7.73)	70 (10.29)	0.038
50 to <60 kg	608 (28.02)	611 (26.85)	204 (30)	
≥60 kg	1,400 (64.52)	1,489 (65.42)	406 (59.71)	
Body mass index, kg/m^2^	25.18 ± 3.39	25.01 ± 3.21	24.9 ± 3.34	0.0887
Baseline SCr, mean SD	1.24 ± 0.18	1.23 ± 0.17	1.28 ± 0.19	<0.001
Baseline SCr, ≥1.5	294 (13.55)	197 (8.66)	150 (22.06)	<0.001
Baseline eGFR, mean SD (mL/min)	54.23 ± 4.11	55.47 ± 3.72	52.21 ± 4.26	<0.001
Follow-up eGFR, mean SD	53.17 ± 4.25	76.16 ± 46.42	37.61 ± 6.44	<0.001
Baseline OAC type				0.004
Warfarin	868 (40)	1,010 (44.38)	304 (44.71)	
Rivaroxaban	521 (24.01)	505 (22.19)	152 (22.35)	
Dabigatran	334 (15.39)	338 (14.85)	88 (12.94)	
Apixaban	318 (14.65)	287 (12.61)	112 (16.47)	
Edoxaban	129 (5.94)	136 (5.98)	24 (3.53)	
Baseline OAC dose				<0.001
Warfarin	868 (40)	1,010 (44.38)	304 (44.71)	
Standard	503 (23.18)	540 (23.73)	106 (15.59)	
Low dose	744 (34.29)	668 (29.35)	245 (36.03)	
Very low dose	55 (2.53)	58 (2.55)	25 (3.68)	
Baseline OAC dose, label				<0.001
Warfarin	868 (40)	1,010 (44.38)	304 (44.71)	
On-label standard dose	451 (20.78)	502 (22.06)	82 (12.06)	
On-label low dose	379 (17.47)	307 (13.49)	121 (17.79)	
Off-label overdose	52 (2.4)	38 (1.67)	24 (3.53)	
Off-label underdose	420 (19.35)	419 (18.41)	149 (21.91)	

CCI, Charlson comorbidity index; COPD, chronic obstructive pulmonary disease; GFR, glomerular filtration rate; OAC, oral anticoagulant; SCr, serum creatinine; SD, standard deviation.

Numbers are mean ± standard deviation or *n* (%).

^a^ The HAS-BLED score was calculated without the use of NSAID due to a lack of information.

### Baseline oral anticoagulant type and dose

The OAC type and dose are presented in Table 1. The proportion of patients who took warfarin in the maintained, improved, and worsened groups were 40.0%, 44.4%, and 44.7%, respectively. The most commonly prescribed DOAC was rivaroxaban in the maintained (24.0%), improved (22.2%), and worsened (16.5%) groups, followed by dabigatran in the maintained (15.4%) and improved (14.9%) groups, and apixaban in the worsened group (16.5%). All groups were mostly prescribed low-dose regimens of the respective DOACs. In case of label adherence of DOAC dosing, the maintained and improved groups had the higher proportions of *on-label standard* doses (20.8% and 22.1%, respectively), while the worsened group was most prescribed off*-label underdosing* (21.9%) at baseline.

### Risk factors of renal function aggravation

Univariable and multivariable analyses were conducted to evaluate the associated factors for renal function aggravation (Supplementary Table S3). On univariable analysis, older age, hypertension, diabetes, heart failure, prior history of MI, PAD, liver disease, COPD, and antiplatelet use were associated with higher risk of renal function aggravation. On multivariable analysis, the presence of hypertension, diabetes, heart failure, and history of MI were significantly associated factors for a higher risk of renal function aggravation—2.0-fold, 1.3-fold, 1.3-fold, and 1.4-fold increase, respectively (Supplementary Table S3).

### Clinical outcomes according to renal function changes

**Figure 2:**
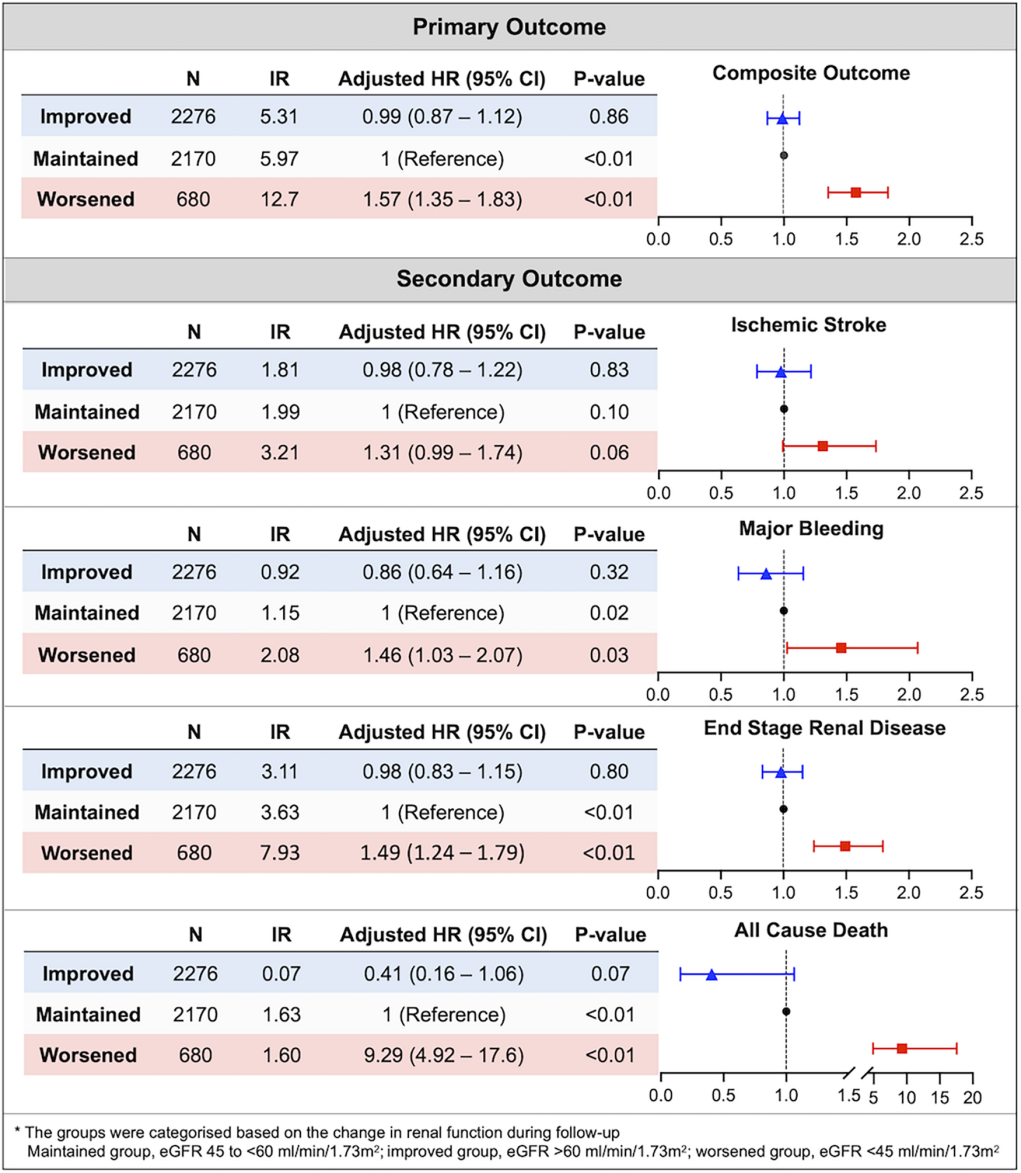
Association between renal function changes during follow-up and the risk of clinical outcomes in patients with marginal renal function (eGFR 45 to <60 ml/min/1.73 m^2^). The aggravation of renal function was significantly associated with a high risk of composite outcome and each component of composite outcome except ischemic stroke. CI, confidence interval; HR, hazard ratio; IR, incidence rate.

The crude event numbers, IR, unadjusted and adjusted HR of primary outcome are presented in Table 2 and Figure 2. Compared to the maintained group, the worsened group was associated with a higher risk of the primary outcome (adjusted HR: 1.574, 95% CI: 1.354–1.830). For the secondary outcomes, the worsened group was associated with significantly higher risks of major bleeding by 46% (adjusted HR: 1.457, 95% CI: 1.027–2.066), mainly driven by hospitalization for GI bleeding (adjusted HR: 1.488, 95% CI: 1.004–2.207). The risk of incident kidney failure was also significantly higher in the worsened group compared to the maintained group (adjusted HR: 1.492, 95% CI: 1.243–1.792). Also, the worsened group showed significantly higher risks of all-cause death, the composite of stroke and major bleeding, and the composite of stroke, major bleeding, and all-cause death.

**Table 2:** Hazard ratios of clinical outcomes according to renal function changes.

	*N*	Event	IR^a^	Model 1 HR (95% CI)	*p*-value	Model 2 HR (95% CI)	*p*-value
Primary outcome							
Stroke + major bleeding + kidney failure + all-cause death							
Maintained	2,170	483	5.97	1 (Reference)		1 (Reference)	
Improved	2,276	462	5.31	0.886 (0.780, 1.007)	0.063	0.988 (0.869, 1.124)	0.860
Worsened	680	284	12.75	2.145 (1.852, 2.484)	<0.001	1.574 (1.354, 1.830)	<0.001
Secondary outcome							
Ischemic stroke							
Maintained	2,170	164	1.99	1 (Reference)		1 (Reference)	
Improved	2,276	161	1.81	0.914 (0.736, 1.137)	0.420	0.976 (0.784,1.216)	0.830
Worsened	680	75	3.21	1.598 (1.216, 2.100)	<0.001	1.311 (0.991, 1.735)	0.058
Intracranial hemorrhage							
Maintained	2,170	24	0.28	1 (Reference)		1 (Reference)	
Improved	2,276	31	0.34	1.214 (0.713, 2.069)	0.475	1.257 (0.733, 2.156)	0.405
Worsened	680	11	0.45	1.585 (0.776, 3.235)	0.206	1.425 (0.687, 2.955)	0.341
GIB							
Maintained	2,170	74	0.88	1 (Reference)		1 (Reference)	
Improved	2,276	53	0.59	0.669 (0.470, 0.952)	0.025	0.736 (0.516, 1.051)	0.092
Worsened	680	40	1.65	1.870 (1.273, 2.748)	0.001	1.488 (1.004, 2.207)	0.048
Major bleeding							
Maintained	2,170	97	1.15	1 (Reference)		1 (Reference)	
Improved	2,276	83	0.92	0.802 (0.598, 1.075)	0.139	0.860 (0.640, 1.156)	0.318
Worsened	680	50	2.08	1.791 (1.273, 2.519)	0.001	1.457 (1.027, 2.066)	0.035
Kidney failure							
Maintained	2,170	311	3.62	1 (Reference)		1 (Reference)	
Improved	2,276	285	3.11	0.851 (0.724, 0.999)	0.048	0.980 (0.833, 1.152)	0.802
Worsened	680	197	7.93	2.217 (1.854, 2.650)	<0.001	1.492 (1.243, 1.792)	<0.001
All-cause death							
Maintained	2,170	14	0.16	1 (Reference)		1 (Reference)	
Improved	2,276	6	0.06	0.396 (0.152, 1.030)	0.057	0.405 (0.155, 1.063)	0.067
Worsened	680	39	1.60	10.087 (5.474, 18.588)	<0.001	9.294 (4.92, 17.555)	<0.001
Stroke + major bleeding							
Maintained	2,170	238	2.94	1 (Reference)		1 (Reference)	
Improved	2,276	235	2.69	0.920 (0.769, 1.102)	0.367	0.990 (0.825, 1.187)	0.910
Worsened	680	122	5.40	1.821 (1.463, 2.265)	<0.001	1.506 (1.204, 1.883)	<0.001
Stroke + major bleeding + all-cause death							
Maintained	2,170	475	5.86	1 (Reference)		1 (Reference)	
Improved	2,276	459	5.26	0.896 (0.788, 1.019)	0.093	1.004 (0.882, 1.143)	0.954
Worsened	680	268	11.86	2.030 (1.748, 2.358)	<0.001	1.478 (1.267, 1.723)	<0.001

CCI, charlson comorbidity index; COPD, chronic obstructive pulmonary disease; DM, diabetes mellitus; DL, dyslipidemia; eGFR, estimated glomerular filtration rate; ESRD, end-stage renal disease; GIB, gastrointestinal bleeding; HF, heart failure; HTN, hypertension; ICH, intracranial hemorrhage; IR, incident rate; MI, myocardial infarction; PAD, peripheral artery disease.

Model 1: Unadjusted.

Model 2: Age, sex, CHA_2_DS_2_-VASc score, HAS-BLED score, CCI, HTN, DM, DL, HF, prior MI, prior Stroke, prior ICH, prior GIB, PAD, Liver disease, COPD, cancer, body weight, eGFR, antiplatelet adjusted.

^a^ IR, per 100 person-years.

Across the primary and secondary outcomes, the improved group did not show statistically significant differences on the risk of clinical outcomes compared to maintained group (Figure 2 and Table 2).

## Discussion

This study has novelty in evaluating the renal function changes in anticoagulated AF patients with marginal renal function (eGFR 45 to <60 ml/min/1.73 m^2^) and clearly demonstrates the association between renal function changes and clinical outcome in this population. Our major findings are as follows: (1) among patients with marginal renal function, a substantial proportion experienced worsening of renal function within 2 years. These patients were older, had lower body weight, and exhibited a higher prevalence of comorbidities; (2) patients with worsened kidney function were often prescribed off-label underdosed DOACs; however, the risk of ischemic stroke did not show a significant difference compared to other groups; and (3) worsening of kidney function within a 2-year period was significantly associated with higher risks of clinical outcomes, especially major bleeding, including hospitalization for GI bleeding, incident kidney failure, and all-cause death.

AF and CKD share common risk factors such as older age, heart failure, and cardiovascular disease (15–17), so they commonly coexist leading to clinically complex phenotypes (18). Indeed, about 40%–50% of AF patients have CKD and 20% of CKD patients have AF (17). CKD may be associated AF substrate changes in the atria, mediated by the activation of inflammation, which is connected to both the structural and electrical remodeling of the atria (19, 20). Also, CKD can be attributed to the activation of the renin-angiotensin-aldosterone system, which is linked to the elevation of atrial pressure, stimulation of atrial fibrosis, and modulation of ion channels within the atria (21, 22). These factors collectively promote the development of AF in patients with CKD.

Impaired renal function in AF patients with anticoagulant is associated with an increased stroke and thromboembolic risk compared to those without renal dysfunction (6, 23). Additionally, impaired renal function is linked to a higher incidence of hemorrhage in elderly AF patients on anticoagulants, with a HR of 1.12 in those aged over 60 and an HR of 2.42 in those aged over 65 (24, 25). Therefore, oral anticoagulants (especially DOACs) should be dose adjusted due to their eliminated to different extents based on renal function. According to current evidence, OACs are considered safe and effective for patients with CKD stages 3a or 3b (26). However, the European guidelines recommend adjusting the dosage of DOACs if the creatinine clearance is below 50 ml/min, due to the association with clinical outcomes (4). The issue is that renal function varies over time, which necessitates appropriate adjustments to the dosing of OACs accordingly. In the ORBIT-AF II registry, changes in renal function among patients with AF were analyzed, around 25% of AF patients showed a reduction of more than 20% in their eGFR during the two-year follow-up period (27). Among the AF patients with a decline in renal function, only one-fifth of those who met the criteria specified in the guidelines for dose reduction were actually prescribed a reduced dosage of DOACs (27). In our study, renal function decline was observed in 13.3% of the cases, and only 3.5% of patients with renal function aggravation were prescribed off-label overdosed OACs at baseline. The differences between previous studies and ours may be attributed to the use of screening data, which likely included a healthier patient population, and the tendency to prescribe off-label underdose regimens in cases of renal function decline among Koreans (28).

According to the guidelines, the dosage of DOACs should be adjusted based on the calculation of creatinine clearance. This is because when using eGFR to estimate renal function, creatinine levels can be influenced by the patient's body weight, age, and sex, leading to potential discrepancies with actual renal function (29). In patients with normal to moderate renal function, there is a possibility that eGFR could underestimate the actual renal function (30), although in actual clinical practice, patient classification based on renal function according to the KDIGO guidelines is determined by eGFR, and patient treatment is conducted accordingly (31).

Through various studies, a decrease in eGFR (<60 ml/min/1.73 m^2^) is associated with an increased risk of cardiovascular disease (32, 33) Indeed, Boriani et al. have previously investigated clinical outcomes based on renal function using eGFR in AF patients (2), and given our study also identified significant adverse clinical outcomes through eGFR, we believe these results can be usefully applied in clinical practice. However, relying solely on eGFR for renal function assessment in AF patients might lead to the underdosing of OAC prescriptions (30, 34).

Renal dysfunction in AF patients is well-established to be associated with adverse clinical outcomes (2, 6, 35, 36). From the ANAFIE registry, a decrease in CrCl was significantly associated with the incidence of stroke, thromboembolic events, clinically relevant nonmajor bleeding, cardiovascular death, and all-cause death (35). A decrease in CrCl or eGFR is as an independent risk factor for adverse clinical outcomes, including stoke and all-cause death (2, 35). Aligned with our study, patient groups exhibiting renal function decline demonstrated a higher risk of major bleeding, all-cause death, and composite outcomes.

To improve outcomes, factors contributing to the deterioration of renal function should be identified and regular renal function assessments made. In AF patients, declining renal function has been associated with advanced age, lower baseline eGFR, coronary artery disease, congestive heart failure, CHA_2_DS_2_-VASc score, and left atrial diameter >45 mm (37–39). Thus AF patients who present with these risk factors along with renal dysfunction need to monitor their renal function frequently. In accordance with the KDIGO guidelines (40), patients with mild to moderate renal function decline, which constituted our initial enrollment population, are advised to undergo renal function assessments 1–3 times per year, depending on the level of albuminuria. One study analyzing AF patients showed that about 60%–80% of patients received regular renal function checks according to the KDIGO guideline (41). Aside from attention to renal function, a holistic or integrated care approach to risk stratification, attention to risk factors and comorbidities is needed for such high risk AF patients (42). This is particularly important given that multimorbidity, polypharmacy and frailty are common, with major implications for outcomes, including bleeding (43–45). The latter is of particular concern in Asian patients and clinical practice (46, 47). Of note, an holistic approach to AF management has been associated with better clinical outcomes (48), leading to its recommendation in guidelines (3).

### Study limitations

This study has several limitations to be acknowledged. First, this study is a retrospective study demonstrating association and causality cannot be deduced. Second, since this study included only the Korean population and we did not verify whether the results could be replicated using an Asian/Korean external validation cohort, there are limitations to the generalizability of the findings. Nevertheless, the novelty of this research lies in its examination of renal function changes and their impact on outcomes, focusing on approximately 5,000 individuals with marginal baseline renal function (eGFR 45 to <60 ml/min/1.73 m^2^) who underwent follow-up examinations. Third, there could be unknown confounders that were not analyzed. Fourth, CKD and renal function decline can have a variety of etiologies, and there is a possibility that some kind of cause of renal function decline might directly increase the risk of primary outcome. However, our study data included baseline and follow-up eGFR values but lacked information regarding the causes of CKD for individual patients, so we could not show detailed causes of CKD and renal function decline. Fifth, this study analyzed a database encompassing the entire Korean population, only a subset undergo regular health examinations provided by the national health system. Additionally, as the study focused on individuals who received follow-up examinations within two years, there may be some selection bias. Sixth, the small numbers preclude detailed comparison of OAC types, given some evidence the renal function decline may be more common in warfarin users compared to DOACs (49). Seventh, information on adherence to OACs and INR levels was not accessible in this database; therefore, we could not analyze the impact of these factors on the study outcomes, which might influence the interpretation of the results. Eighth, the follow-up duration of our study was relatively short [median 0.81 years (IQR: 0.17–2.19) and mean 1.37 ± 1.45 years]. Although the median follow-up in previously reported observational studies of clinical outcomes in patients with anticoagulated AF is often less than 1 year, longer follow-up analyses would allow us to analyze the risk of long-term clinical outcomes with changes in renal function. In our study, the finding that renal function changes were associated with short or mid-term clinical outcomes should be interpreted with caution. Ninth, our study excluded patients lacking pre-anticoagulation laboratory tests, as baseline renal function could not be assessed, which may introduce selection bias by excluding those with severe illnesses who could not be screened. Tenth, although AF type and actual burden can affect the risk of renal function changes over time and the occurrence of clinical outcomes (50), our database did not contain information regarding the type and burden of AF.

## Conclusion

In anticoagulated AF patients with marginal renal function, a substantial proportion of patients experienced renal function decline below eGFR 45 ml/min/1.73 m^2^ within 2 years. Renal function decline was associated with higher risks of major bleeding, ESRD, all-cause death, and the composite outcome compared to those who maintained their baseline renal function. Regular checks and review of renal function should be emphasized in AF patients with marginal renal function.

## Data Availability

Publicly available datasets were analyzed in this study. This data can be found here: https://www.hira.or.kr/main.do.
