# Supplementary material for: Association between changes in renal function and clinical outcomes in anticoagulated atrial fibrillation patients with marginal renal function. A nationwide observational cohort study
Source: Front Cardiovasc Med. 2024 Jun 6;11:1423336. doi: 10.3389/fcvm.2024.1423336 (PMC11188773; doi:10.3389/fcvm.2024.1423336)
Supplement: Supplementary file 1 [file Table1.docx]

**Association between changes in renal function and clinical outcomes in anticoagulated atrial fibrillation patients with marginal renal function. A nationwide observational cohort study**

Kyung-Yeon Lee, MD^1^*, So-Ryoung Lee, MD, PhD^1,2^*, Eue-Keun Choi, MD, PhD^1,2^, JungMin Choi, MD^1^, Hyo-Jeong Ahn, MD^1^, Soonil Kwon, MD^3^, Bongseong Kim^4^, Kyung-Do Han^4^, Seil Oh, MD, PhD^1,2^, Gregory Y. H. Lip, MD^2,5,6^

^1^Department of Internal Medicine, Seoul National University Hospital, Seoul, Republic of Korea

^2^Department of Internal Medicine, Seoul National University College of Medicine, Seoul, Republic of Korea

^3^Division of Cardiology, Department of Internal Medicine, Boramae Medical Center, Seoul National University College of Medicine, Seoul, Korea.

^4^Statistics and Actuarial Science, Soongsil University, Seoul, Republic of Korea

^5^Liverpool Centre for Cardiovascular Science at University of Liverpool, Liverpool John Moores University and Liverpool Chest & Heart Hospital, Liverpool, United Kingdom

^6^Danish Center for Health Services Research, Department of Clinical Medicine, Aalborg University, Aalborg, Denmark

**Supplementary Tables**

**Table S1. Definitions of covariates and study outcomes**

**Table S2. Charlson comorbidity index**

**Table S3. Comparison between univariable model and multivariable model for risk of renal function aggravation**

**Table S1. Definitions of covariates and study outcomes**

| **Diagnosis** | **ICD-10-CM code and definition** | **Diagnostic definition** |
| --- | --- | --- |
| **Inclusion/exclusion criteria** |  |  |
| **Atrial fibrillation** | I48.0-48.4, I48.9 | Admission or outpatient department≥1 |
| **Valvular atrial fibrillation** | I05.0, I05.2, I05.9, Z95.2-Z95.4 | Admission or outpatient department≥1 |
| **Comorbidities** |  |  |
| **Hypertension** | I10-I13, I15; and minimum 1 prescription of anti-hypertensive drug (thiazide, loop diuretics, aldosterone antagonist, alpha-/beta-blocker, calcium-channel blocker, angiotensin-converting enzyme inhibitor, angiotensin II receptor blocker). | Admission≥1 or outpatient department≥1 |
|  | Or systolic/diastolic blood pressure ≥ 140/90 mmHg | Index health examination |
| **Diabetes mellitus** | E11-E14; and minimum 1 prescription of anti-diabetic drugs (sulfonylureas, metformin, meglitinides, thiazolidinediones, dipeptidyl peptidase-4 inhibitors, α-glucosidase inhibitors, and insulin). | Admission≥1 or outpatient department≥1 |
|  | Or fasting glucose level ≥ 126 mg/dL | Index health examination |
| **Dyslipidemia** | E78 | Admission or outpatient department≥1 |
|  | Or Total cholesterol ≥ 240 mg/dL | Index health examination |
| **Heart failure** | I50 | Admission or outpatient department≥1 |
| **Prior ischemic stroke** | I63, I64 | Admission or outpatient department≥1 |
| **Prior intracranial hemorrhage** | I60, I61, I62 | Admission or outpatient department≥1 |
| **Prior myocardial infarction** | I21, I22 | Admission or outpatient department≥1 |
| **Peripheral artery disease** | I70, I73 | Admission or outpatient department≥1 |
| **Chronic obstructive pulmonary disease** | J41-44 | Admission or outpatient department≥1 |
| **Cancer** | C00-97 and RID code (V193) | Admission or outpatient department≥1 |
| **Scores** |  |  |
| **CHA_2_DS_2_-VASc score** | Heart failure (1 point), hypertension (1 point), age ≥75 years (2 points), diabetes (1 point), previous stroke/systemic embolism/transient ischemic attack (2 points), vascular disease (prior MI or PAD, 1 point) and female sex (1 point) | |
|  |  |  |
| **Clinical outcome** |  |  |
| **Ischemic stroke** | I63, I64 | Primary diagnosis, admission≥1 (≥3 days) and brain imaging (CT or MRI) ≥1 |
| **Myocardial infarction** | I21, I22 | Primary diagnosis, admission≥1 |
| **Hospitalization for heart failure** | I50 | Primary diagnosis, admission≥1 |
| **kidney failure** | N18.5, Z49 | Dialysis ≥2  Dialysis: hemodialysis (O7011-O7020), or peritoneal dialysis (O7017, O7075), Kidney transplantation (R3280) |
| **Composite outcomes** | Ischemic stroke + major bleeding + kidney failure + all-cause death | Each definition was described as above. |

**Table S2. Charlson comorbidity index**

| **Category** | **Weights** | **Disease** | **ICD-10-CM code** |
| --- | --- | --- | --- |
| **Myocardial infarction** | 1 | Acute myocardial infarction | I21 |
|  |  | Subsequent myocardial infarction | I22 |
| **Congestive heart failure** | 1 | Heart Failure | I50 |
| **Peripheral vascular disease** | 1 | Atherosclerosis | I70 |
|  |  | Other peripheral vascular disease | I73 |
| **Cerebrovascular disease** | 1 | Transient cerebral ischemic attacks and related syndromes | G45 |
|  |  | Vascular syndromes of brain in cerebrovascular diseases | G46 |
|  |  | Retinal vascular occlusion | H34 |
|  |  | Cerebrovascular disease | I60-I69 |
| **Dementia** | 1 | Dementia in Alzheimer disease | F00 |
|  |  | Vascular dementia | F01 |
|  |  | Dementia in other disease classified elsewhere | F02 |
|  |  | Unspecified dementia | F03 |
| **Chronic pulmonary disease** | 1 | Chronic lower respiratory diseases | J40-J47 |
|  |  | Lung disease due to external agents | J60-J67 |
| **Rheumatic disease**  **(connective tissue disorder)** | 1 | Rheumatoid arthritis with rheumatoid factor | M05 |
|  |  | Felty's syndrome | M05.0 |
|  |  | Rheumatoid lung disease with rheumatoid arthritis | M05.1 |
|  |  | Rheumatoid vasculitis with rheumatoid arthritis | M05.2 |
|  |  | Rheumatoid heart disease with rheumatoid arthritis | M05.3 |
|  |  | Rheumatoid myopathy with rheumatoid arthritis | M05.4 |
|  |  | Rheumatoid polyneuropathy with rheumatoid arthritis | M05.5 |
|  |  | Rheumatoid arthritis with involvement of other organs and systems | M05.6 |
|  |  | Rheumatoid arthritis with rheumatoid factor without organ or systems involvement | M05.7 |
|  |  | Other rheumatoid arthritis with rheumatoid factor | M05.8 |
|  |  | Rheumatoid arthritis without rheumatoid factor | M05.9 |
|  |  | Adult-onset Still's disease | M06.1 |
|  |  | Rheumatoid bursitis | M06.2 |
|  |  | Rheumatoid nodule | M06.3 |
|  |  | Inflammatory polyarthropathy | M06.4 |
|  |  | Other specified rheumatoid arthritis | M06.8 |
|  |  | Rheumatoid arthritis, unspecified | M06.9 |
|  |  | Giant cell arteritis with polymyalgia rheumatica | M31.5 |
|  |  | Systemic lupus erythematosus (SLE) | M32 |
|  |  | Drug-induced SLE | M32.0 |
|  |  | SLE with organ or system involvement | M32.1 |
|  |  | Other forms of SLE | M32.8 |
|  |  | SLE, unspecified | M32.9 |
|  |  | Dermatopolymyositis | M33 |
|  |  | Juvenile dermatomyositis | M33.0 |
|  |  | Other dermatomyositis | M33.1 |
|  |  | Polymyositis | M33.2 |
|  |  | Dermatopolymyositis, unspecified | M33.9 |
|  |  | Systemic sclerosis [scleroderma] | M34 |
|  |  | Progressive systemic sclerosis | M34.0 |
|  |  | CR(E)ST syndrome | M34.1 |
|  |  | Systemic sclerosis induced by drug and chemical | M34.2 |
|  |  | Other forms of systemic sclerosis | M34.8 |
|  |  | Systemic sclerosis, unspecified | M34.9 |
|  |  | Other overlap syndromes | M35.1 |
|  |  | Polymyalgia rheumatica | M35.3 |
|  |  | Dermato(poly)myositis in neoplastic disease | M36.0 |
| **Peptic ulcer disease** | 1 | Gastric ulcer | K25 |
|  |  | Duodenal ulcer | K26 |
|  |  | Peptic ulcer, site unspecified | K27 |
|  |  | Gastrojejunal ulcer | K28 |
| **Mild liver disease** | 1 | Chronic viral hepatitis | B18 |
|  |  | Alcoholic fatty liver | K70.0- K70.3, K70.9 |
|  |  | Alcoholic hepatitis |  |
|  |  | Alcoholic fibrosis and sclerosis of liver |  |
|  |  | Alcoholic cirrhosis of liver |  |
|  |  | Alcoholic liver disease, unspecified |  |
|  |  | Toxic liver disease with chronic persistent hepatitis | K71.3- K71.5, K71.7 |
|  |  | Toxic liver disease with chronic lobular hepatitis |  |
|  |  | Toxic liver disease with chronic active hepatitis |  |
|  |  | Toxic liver disease with fibrosis and cirrhosis of liver |  |
|  |  | Chronic hepatitis, not elsewhere classified | K73 |
|  |  | Fibrosis and cirrhosis of liver | K74 |
|  |  | Fatty (change of) liver, not elsewhere classified | K76.0-K76.4, K76.8, K76.9 |
|  |  | Nonalcoholic fatty liver disease |  |
|  |  | Central hemorrhagic necrosis of liver |  |
|  |  | Infarction of liver |  |
|  |  | Hepatic angiomatosis |  |
|  |  | Other specified disease of liver |  |
|  |  | Simple cyst of liver |  |
|  |  | Focal nodular hyperplasia of liver |  |
|  |  | Hepatoptosis |  |
|  |  | Liver disease, unspecified |  |
|  |  | Liver transplant status | Z94.4 |
| **Diabetes without chronic**  **complication** | 1 | with coma | E10.0, 10.1, 10.6, 10.8, 10.9 |
|  |  | with ketoacidosis | E11.0, 11.1, 11.6, 11.8, 11.9 |
|  |  | with other specified complications | E12.0, 12.1, 12.6, 12.8, 12.9 |
|  |  | with unspecified complications | E13.0, 13.1, 13.6, 13.8, 13.9 |
|  |  | without complications | E14.0, 14.1, 14.6, 14.8, 14.9 |
| **Diabetes with chronic**  **complication** | 2 | with renal complications | E10.2, 10.3, 10.4, 10.5, 10.7 |
|  |  | with ophthalmic complications | E11.2, 11.3, 11.4, 11.5, 11.7 |
|  |  | with neurologic complications | E12.2, 12.3, 12.4, 12.5, 12.7 |
|  |  | with peripheral circulatory complications | E13.2, 13.3, 13.4, 13.5, 13.7 |
|  |  | with multiple complications | E14.2, 14.3, 14.4, 14.5, 14.7 |
| **Hemi/paraplegia** | 2 | Tropical spastic paraplegia | G04.1 |
|  |  | Hereditary spastic paraplegia | G11.4 |
|  |  | Spastic quadriplegic cerebral palsy | G80.0 |
|  |  | Spastic diplegic cerebral palsy | G80.1 |
|  |  | Spastic hemiplegic cerebral palsy | G80.2 |
|  |  | Flaccid hemiplegia | G81.0 |
|  |  | Spastic hemiplegia | G81.1 |
|  |  | Hemiplegia, unspecified | G81.9 |
|  |  | Flaccid paraplegia | G82.0 |
|  |  | Spastic paraplegia | G82.1 |
|  |  | Paraplegia, unspecified | G82.2 |
|  |  | Flaccid tetraplegia | G82.3 |
|  |  | Spastic tetraplegia | G82.4 |
|  |  | Tetraplegia, unspecified | G82.5 |
|  |  | Diplegia of upper limbs | G83.0 |
|  |  | Paralytic syndrome, unspecified | G83.9 |
| **Renal disease** | 2 | Hypertensive renal disease | I12 |
|  |  | Hypertensive heart and renal disease with renal failure | I13.1 |
|  |  | Chronic nephritic syndrome | N03 |
|  |  | Unspecified nephritic syndrome | N05 |
|  |  | Chronic kidney disease | N18 |
|  |  | Unspecified kidney failure | N19 |
|  |  | Disorders resulting from impaired renal tubular function | N25 |
|  |  | Care involving dialysis | Z49 |
|  |  | Transplanted organ and tissue status - kidney | Z94.0 |
|  |  | Dependence on renal dialysis | Z99.2 |
| **Cancer** | 2 | Any tumor, malignant neoplasm | C00-76, C97 |
|  |  | Any tumor, in situ neoplasm | D00-09 |
|  |  | Any tumor, Benign neoplasm | D10-36 |
|  |  | Any tumor, Neoplasm of unknown behavior | D37-48 |
|  |  | Leukemia | C91-95 |
|  |  | Lymphoma | C81-86 |
| **Metastatic cancer** | 3 | Metastatic solid tumor | C77-80 |
| **Moderate to severe**  **liver disease** | 3 | Esophageal varices | I85 |
|  |  | Gastric varices | I86.4 |
|  |  | Esophageal varices without bleeding in diseases classified elsewhere | I98.2 |
|  |  | Alcoholic hepatic failure | K70.4 |
|  |  | Toxic liver disease with hepatic necrosis | K71.1 |
|  |  | Hepatic failure (acute/chronic) due to drugs |  |
|  |  | Chronic hepatic failure | K72.1, K72.9 |
|  |  | Hepatic failure, unspecified |  |
|  |  | Hepatic veno-occlusive disease | K76.5-K76.7 |
|  |  | Portal hypertension |  |
|  |  | Hepatorenal syndrome |  |
| **Human immunodeficiency**  **Virus (HIV)** | 6 | HIV disease resulting in infectious and parasitic diseases | B20 |
|  |  | HIV disease resulting in malignant neoplasm | B21 |
|  |  | HIV disease resulting in other specified diseases | B22 |
|  |  | HIV disease resulting in other conditions | B23 |

**Table S3. Comparison between univariable model and multivariable model for risk of renal function aggravation**

|  | **Univariate** | | **Multivariate** | |
| --- | --- | --- | --- | --- |
|  | **OR (95% CI)** | **P-value** | **OR (95% CI)** | **P-value** |
| **Age, year** | 1.071 (1.059, 1.084) | <0.001 | 1.067 (1.053, 1.08) | <0.001 |
| **Hypertension** | 2.524 (1.595, 3.996) | <0.001 | 1.999 (1.254, 3.186) | 0.004 |
| **Diabetes mellitus** | 1.551 (1.313, 1.832) | <0.001 | 1.338 (1.12, 1.599) | 0.001 |
| **Dyslipidemia** | 1.233 (0.99, 1.536) | 0.062 | 1.046 (0.828, 1.322) | 0.704 |
| **Heart failure** | 1.439 (1.22, 1.698) | <0.001 | 1.255 (1.057, 1.489) | 0.001 |
| **Prior MI** | 1.682 (1.238, 2.287) | 0.001 | 1.407 (1.023, 1.934) | 0.036 |
| **Peripheral artery disease** | 1.252 (1.058, 1.481) | 0.009 | 1.049 (0.881, 1.25) | 0.591 |
| **Liver disease** | 1.22 (1.037, 1.435) | 0.016 | 1.106 (0.933, 1.311) | 0.245 |
| **COPD** | 1.578 (1.221, 2.039) | 0.001 | 1.146 (0.878, 1.496) | 0.316 |
| **Antiplatelet use** | 1.303 (1.106, 1.535) | 0.002 | 1.187 (1.002, 1.407) | 0.048 |
| **Baseline body weight** | 0.985 (0.978, 0.993) | <0.001 | 0.999 (0.991, 1.007) | 0.768 |

Abbreviations: MI, myocardial infarction; COPD, chronic obstructive pulmonary disease.
